# Surface Engineering of Regenerated Cellulose Nanocomposite Films with High Strength, Ultraviolet Resistance, and a Hydrophobic Surface

**DOI:** 10.3390/polym15061427

**Published:** 2023-03-14

**Authors:** Ying Zhu, Tianhao Wang, Yanan Dai, Ye Wang, Yukun Ding, Liping Zhang

**Affiliations:** Department of Chemistry and Chemical Engineering, MOE Engineering Research Center of Forestry Biomass Materials and Energy, Beijing Forestry University, Beijing 100083, China; lingong16zhuying@bjfu.edu.cn (Y.Z.);

**Keywords:** regenerative cellulose film, octadecyltrichlorosilane, surface silanization modification, hydrophobic surface, ultraviolet blocking

## Abstract

Regenerated cellulose packaging materials can alleviate the environmental pollution and carbon emissions caused by conventional plastics and other chemicals. They require regenerated cellulose films with good barrier properties, such as strong water resistance. Herein, using an environmentally friendly solvent at room temperature, a straightforward procedure for synthesizing these regenerated cellulose (RC) films, with excellent barrier properties and doping with nano-SiO_2_, is presented. After the surface silanization modification, the obtained nanocomposite films exhibited a hydrophobic surface (HRC), in which the nano-SiO_2_ provided a high mechanical strength, whereas octadecyltrichlorosilane (OTS) provided hydrophobic long-chain alkanes. The contents of the nano-SiO_2_ and the concentrations of the OTS/*n*-hexane in regenerated cellulose composite films are crucial, as they define its morphological structure, tensile strength, UV-shielding ability, and the other performance of these composite films. When the nano-SiO_2_ content was 6%, the tensile stress of the composite film (RC6) increased by 41.2%, the maximum tensile stress was 77.22 MPa, and the strain-at-break was 14%. Meanwhile, the HRC films had more superior multifunctional integrations of tensile strength (73.91 MPa), hydrophobicity (HRC WCA = 143.8°), UV resistance (>95%), and oxygen barrier properties (5.41 × 10^−11^ mL·cm/m^2^·s·Pa) than the previously reported regenerated cellulose films in packaging materials. Moreover, the modified regenerated cellulose films could biodegrade entirely in soil. These results provide an experimental basis for preparing regenerated-cellulose-based nanocomposite films that exhibit a high performance in packaging applications.

## 1. Introduction

Our daily life is filled with plastic bags and other products made of plastic; however, traditional petroleum-based plastic products put a huge strain on the environment [1]. To address the substantial pollution that corresponds to plastic waste, the ecologically friendly materials made from natural polymers or produced from renewable resources were a focus of the current study [2,3].

Cellulose is one of nature’s most common polymers. Cellulose and its derivatives are biocompatible and are slowly degraded [4,5]. Because of its excellent biocompatibility and low costs, many applications use cellulose. Regenerated cellulose films are suitable packaging materials. However, using RC films to meet market demands is difficult because of the films’ low strength, water resistance, and gas barrier at high humidities. The development of regenerated cellulose packaging films with superior multifunctional integrations of strength, barrier performance, and hydrophobicity can help to alleviate the environmental pollution and carbon emissions that correspond to traditional plastics [6,7]. 

The chemical composition and micro–nano structure of a material’s surface have a strong influence on its wettability [8]. The researchers generated novel surfaces with micro- and nanostructures (through various strategies) and low surface energy by researching the surfaces of rice leaves, lotus leaves, and butterfly wings. The main methods currently employed for the hydrophobic alteration of cellulose surfaces are surface structures [9], esterification grafting [10], long-chain alkane grafting [11], surface hydrophobic coatings [12], and silylation grafting, among others. For example, silylation grafting, hydrophobically, can be used to modify the surface of solid cellulose materials such as cellulose aerogels [13], yet few studies have focused on the hydrophobic modification of cellulose film surfaces using this method. OTS is a well-known organosilane derivative. This commercially available chemical is widely recognized for changing the surface characteristics of diverse solid substrates by creating a compact and strongly aligned self-assembled monolayer [14]. Its fluorine-free composition reduces environmental and health risks [15]. Anuj Kumar et al. used octadecyl trichlorosilane (OTS) for the surface modification of wood fibrous insulators to obtain a hydrophobic surface and improve their effectiveness towards mold significantly [16]. KehaoFan et al. used a 1% octadecyl trichlorosilane (OTS)/*n*-hexane solution for a functionalized hydrophobic modification of regenerated cellulose aerogels. This study found that octadecyl trichlorosilane covered the entire fiber surface and constructed a hydrophobic surface, the contact angle increased with the increase of the octadecyl trichlorosilane content, and the microstructure of the cellulose aerogel was not changed [17]. Grafting on the surface of an RC film with octadecyl trichlorosilane can make it hydrophobic. 

Due to its high fraction of intra- and intermolecular hydrogen bonding, cellulose is hard to treat in solution or as a melt. The solvent quaternary ammonium ionic liquids (ILs) that were developed in the present authors’ laboratory can dissolve cellulose at low temperatures; a series of regenerated cellulose functional fibers have exhibited good properties in previous reports [18,19]. Using blend modification and adding additional fillers enhances the performance of the RC film, rendering the film more widely useful. Fillers such as metallic oxide nanoparticles (e.g., ZnO, TiO_2_ and Al_2_O_3_) can impart excellent gas barrier properties and UV-blocking capability to polymers, but their dispersibility in composites is low [20]. Nano-SiO_2_ has the following characteristics: a low cost, low toxicity, good stability, low surface energy, and is fully mixable with cellulose in this ILs system. Thus, nano-SiO_2_ is an ideal cellulose nanofiller. To the best of our knowledge, no one has documented the application of the hydrophobic alteration of RC films using nano-SiO_2_ and OTS to create waterproof packaging materials.

In this study, the hydrophobic nanocomposite films were obtained by preparing a film via immersion precipitation phase inversion, dissolving cellulose pulp in an IL solvent system, introducing nano-SiO_2_, and modifying the surface silanization using OTS to obtain an HRC film, which has more superior multifunctional integrations of tensile strength, strain-at-break, hydrophobicity, UV resistance, and oxygen barrier properties than the previously reported regenerated cellulose films in packaging materials [21]. Moreover, the modified regenerated cellulose films biodegrade entirely in soil. Such films can help to replace some of the petroleum-based plastics and have substantial potential for applications as waterproof packaging materials.

## 2. Materials and Methods

### 2.1. Materials

Senbo provided the hardwood pulpboard (degree of polymerization: 750) (Shandong, China). Shanghai Macklin Biochemical supplied the hydrophobic SiO_2_ nanoparticles, OTS, and DMSO (Shanghai, China). The primary particles of the SiO_2_ nanoparticles were 15 nm in diameter and had hydrophobic silanol (Si–CH_3_) groups attached to their surfaces. The *n*-Hexane was purchased from Concord Technology (Tianjin, China). The trials were conducted using deionized water.

### 2.2. Method

#### 2.2.1. Preparation of Nano-SiO_2_/Cellulose Solutions

To eliminate moisture, the cellulose pulp was dried at 60 °C for 12 h. The nano-SiO_2_ was mixed with the laboratory-made IL in a round-bottomed flask and sonicated for 1 h to obtain a homogeneous mixture [18]. Then, the cellulose pulp was added to the nano-SiO_2_/IL solution via mechanical stirring at 55 °C for 4 h, in order to prepare the nano-SiO_2_/cellulose solutions with a cellulose concentration of 8% and nano-SiO_2_ contents of 0, 2, 4, 6, 8, and 10 wt% of cellulose content. To eliminate impurities and bubbles, the nano-SiO_2_/cellulose solution was centrifuged at 1700× *g* for 20 min until the solution was optically homogeneous. The solutions were sealed and kept in a P_2_O_5_-containing desiccator.

#### 2.2.2. Preparation of Nano-SiO_2_/Regenerated Cellulose Composite Films

The regenerated cellulose composite films were prepared by immersion precipitation phase inversion. An amount of the homogeneous mixed solution was applied to the glass plates at 55 °C, scraped with a 550-μm-thick applicator to form a semi-dry gelation, and then regenerated in a coagulation bath (volume ratio deionized water: DMSO = 7:3) at 25 °C. The finished hydrogels were immediately solidified in the deionised water for 4 h to remove the ionic liquid before being plasticized in glycerol. To avoid wrinkling and to further consolidate the SiO_2_ arrangement, the treated films were placed on acrylic sheets and then dried in an electric air oven at 50 °C for 4 h. The films were coded as RC0, RC2, RC4, RC6, RC8, and RC10, according to the mass percent of the nano-SiO_2_.

#### 2.2.3. Hydrophobic Modification of Regenerated Cellulose Composite Films

For the preparation of the hydrophobic modifiers, 0.25 mL of OTS was suspended in 50 mL of *n*-hexane and ultrasonicated for 30 min. The hydrophobic regenerated cellulose film (HRC) was obtained via an in situ redistribution infiltration into sample RC6, followed by being washed 3× with *n*-hexane and dried for 1 h at room temperature. The volume ratios of OTS and *n*-hexane were 0.5/100, 1/100, 1.5/100, 2/100, 2.5/100, and 3/100. The hydrophobic RC6 films with various concentrations of OTS were coded as HRC0.5, HRC1, HRC1.5, HRC2, HRC2.5, and HRC3.

#### 2.2.4. Characterization

The rheological properties of the cellulose solutions were measured on the rotational rheometer (CVO-100, Bohlin, Malvern, UK). The cellulose solution was poured onto the rheometer platform, and the cellulose solutions’ static rheological characteristics were examined using a cone clamp (diameter: 40 mm; cone angle: 2°). The shear rate ranged from 0.1–100 s^−1^.

The existence of functional groups in the nano-SiO_2_/regenerated cellulose composite films was examined using an FTIR spectrometer. (FTIRPE-2000, Perkin–Elmer, Waltham, MA, USA) in the range of 500–4000 cm^−1^ at a resolution of 4 cm^−1^ (with a KBr pellet).

To study the effect of the nano-SiO_2_ and OTS on the crystallinity of the RC films, XRD measurements were performed (Bruker D8 Advance, Brook, Germany) at 40 kV and 100 mA with Cu–Kα radiation [22].

The solid-state ^13^C-NMR spectra of the samples were determined using a Bruker 400 M NMR spectrometer at 25 °C. The MAS spin rate was: 10 Khz; recovery time: 4 s; pulse program for acquisition: cp; and pre-scan delay: 6.5 μs.

SEM (S-3000N, Hitachi, Tokyo, Japan) was used to characterize the cross-sections and surfaces of the films. Energy-dispersive X-ray spectroscopy (EDS) was used to create elemental and distribution maps of the nano-SiO_2_ and OTS in the fiber cross-sections from the SEM data. The dispersion state of the nano-SiO_2_ within the cellulose was investigated using transmission electron microscopy (TEM; JEM-1010, Tokyo, Japan) at an accelerating voltage of 300 kV.

The decomposition behaviors of the composite films were analyzed with a TGA (TGA-600, Shimadzu, Japan) machine, over a temperature range of 30–800 °C, under a nitrogen atmosphere, and at a constant heating rate of 20 °C/min. A differential thermal analysis (DTA) was also used to determine the enthalpy change of the films as the function of the temperature.

The tensile strength of the films was measured with a universal testing machine (Instron 5848, Norwood, MA, USA), at a crosshead speed of 10 mm/min and a gauge length of 100 mm. The thickness of the film was measured with a helical micrometer (XPV-25, Pudan, Shanghai, China). The tensile strength and elongation were estimated as the averages of at least five stress–strain curve measurements.

The UV–vis spectra of the sample films were collected with a UV–vis spectrophotometer (PerkinElmer Lambda 650, Waltham, MA, USA) over the range of 200–1000 nm. Before the UV–vis measurements were taken, the films were adhered to the surface of the quartz pool.

A contact angle goniometer (JGW-360a, HAKE, Beijing, China) was used to test the contact angle of the film surface at room temperature. The static contact angle of the samples was measured using water as the measurement droplet in a volume of 10 μL. In total, five different locations were selected on each sample to measure the water contact angle, and the average of the five contact angles was taken as the final measurement result to guarantee data accuracy and reproducibility. The equilibrium state was determined when the droplet size and contact angle did not change within 10 min; the sampling error was ±1.5%.

To evaluate the composite films’ water resistance, the dried RC films were weighed (*m*_0_) and immersed in deionized water in flasks, and taken out at regular intervals; the water that adhered to the film surface was absorbed with filter paper and the films were weighed again (*m*_t_). The water absorption percent (*W*%) of the film was calculated (Equation (1)). A total of three measurements were taken for each sample to guarantee data accuracy and repeatability.

(1) $$W\left(\%\right)=\left(m_{t}-m_{0}\right)/m_{0}$$

The oxygen permeability of the composite films was conducted on a 10 cm × 10 cm surface sample with a VAC-V1 permeability analyzer, in accordance with the standard GB/T 1038–2000. The thickness of the samples was measured with a micrometer caliper.

## 3. Results and Discussion

### 3.1. Morphology and Structure of the Films

**Figure 1:**
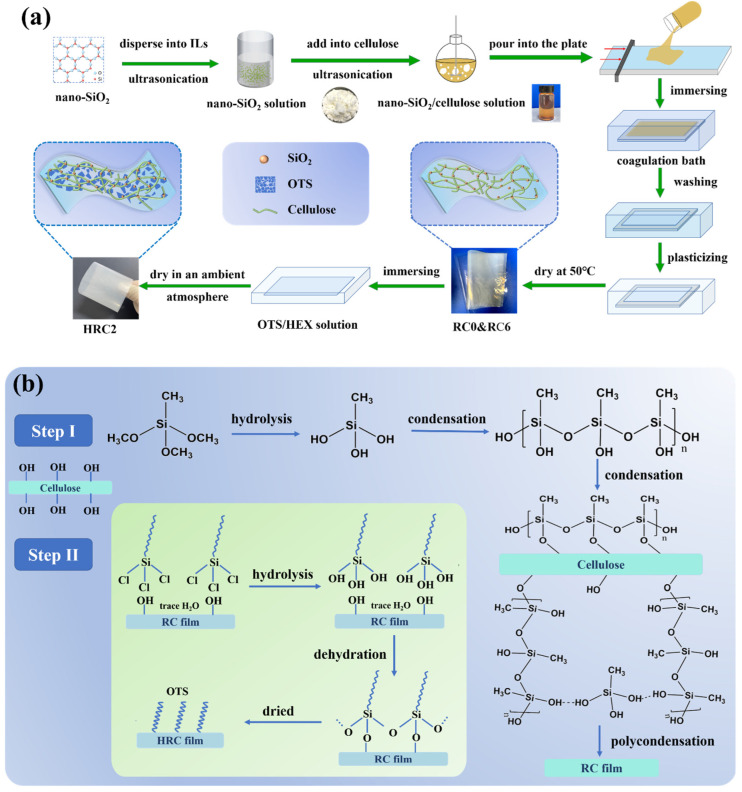
(**a**) Schematic for preparing regenerated cellulose composite films, and (**b**) schematic diagram of the mechanism of RC and HRC films during the forming process.

Figure 1 shows a schematic reaction route for the preparation of RC and HRC films. In this study, hydrophobic regenerated cellulose composite films were prepared in a two-step process. In step Ⅰ, the condensation of silane takes place under alkaline condensation and the nano-SiO_2_ particles are dispersed in the presence of a silane coupling agent, and then react with cellulose after hydrolysis and condensation under acidic conditions. The diagram shows how nano-SiO_2_ is complex in cellulose, that is, the route by which the nano-SiO_2_ particles are then grown in situ on the regenerated cellulose film [23,24,25]. Step Ⅱ shows the interactions between the OTS and the nano-SiO_2_/regenerated cellulose composite film surface. First, the OTS hydrolyzes with the trace water on the surface of the substrate, in a manner that converts Si–Cl bonds into Si-OH bonds, then the Si-OH bonds react with –OH bonds (by dehydration), and finally, the OTS alkyl chains form covalent bonds, thus resulting in modification. The OTS provides hydrophobic long-chain alkanes, whereas nano-SiO_2_ gives a high mechanical strength. OTS crystals are evenly distributed onto the surface of the cellulose films after solvent-vaporized crystallization, resulting in a micro–nano binary structure and interstitial spaces between the microplates, affecting the hydrophobicity and self-cleaning properties of the RC films. At the same time, the surface-modified HRC films exhibit remarkable UV-shielding capability and oxygen barrier performance. Such properties might be useful in next-generation waterproof packaging [26,27].

**Figure 2:**
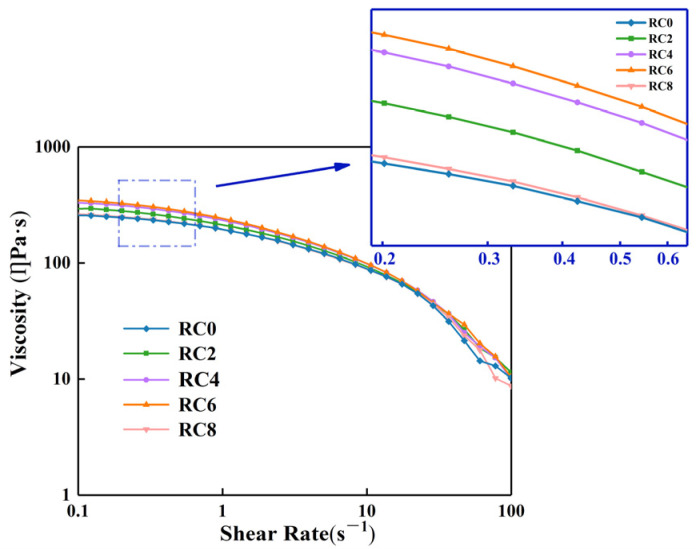
Rheological properties of composite film liquids with various quantities of added nano-SiO_2_.

The viscosity of the cellulose solution substantially influenced the scraping and the resultant RC film morphology. Figure 2 depicts the change in the cellulose solution viscosity, with an increasing shear rate for the solutions with various nano-SiO_2_ levels. Shear-thinning was seen in all the cellulose solutions, with and without nano-SiO_2_, which is a notable property of macromolecular solutions [28]. The characteristics of the cellulose solution that was created after blending with nano-SiO_2_ were comparable to those of a conventional polymer solution, implying that the cellulose solution remained stable after adding nano-SiO_2_. The findings also suggest that the zero-shear viscosity of the cellulose solution rose progressively as the nano-SiO_2_ concentration grew from 2–6 wt%. The high surface energy of the SiO_2_ facilitated the adsorption of cellulose macromolecules in a manner that resulted in physical crosslinking points, which increased the entanglement between the cellulose macromolecules and increased the polymer viscosity to a higher value than that of the RC0 film liquid. Over the range of 6–8 wt%, the viscosity of the composite film solution decreased because of the uneven dispersion of the SiO_2_ nanoparticles and agglomerations, but the zero-cut viscosity of the RC films was higher than that of the RC0 film solution [29]. This result was verified by a mechanical analysis (discussed in subsequent paragraphs).

**Figure 3:**
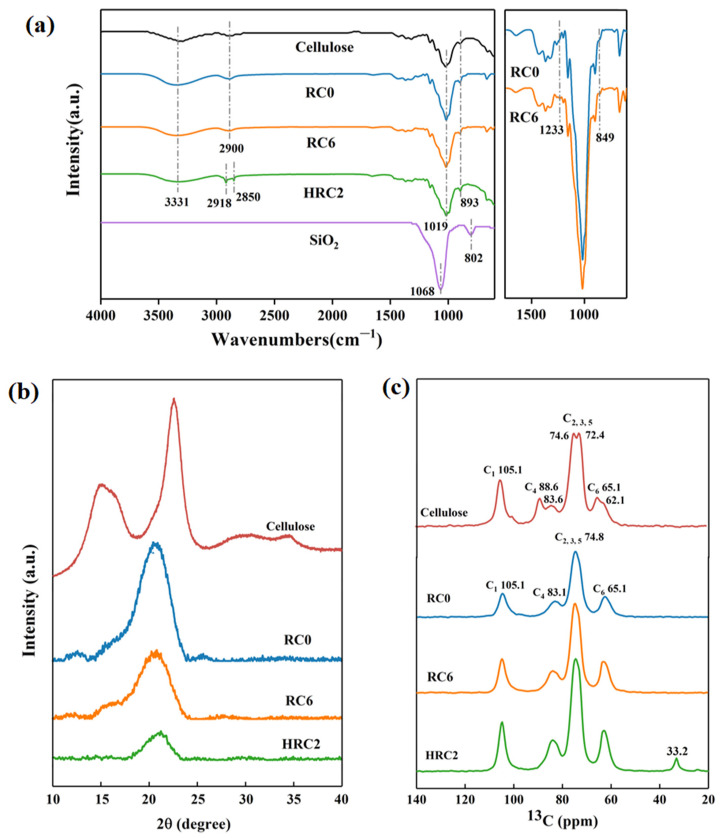
(**a**) FTIR spectra of various samples, and FTIR spectra of RC0 and RC6 films, (**b**) XRD curves, and (**c**) the CP/MAS ^13^C-NMR spectra of various samples.

Figure 3a shows the FTIR spectra of the cellulose, RC0, RC6, HRC2, and pure nano-SiO_2_. The double peaks of the wood pulp cellulose between 1500 and 1300 cm^−1^ are C–H bending vibrations, and those <910 cm^−1^ are the C–H bending vibrations of the aromatic hydrocarbons. The absorption peaks of the infrared spectrum of the RC0 film are in the infrared spectrum of the RC6 film, as are the absorption peaks at 3100–3600 cm^−1^. The latter is the absorption peak of the stretching vibration of the hydrogen bond –OH. The absorption peaks at 2870–2960 cm^−1^ are the symmetrical stretching vibration of C–H, the absorption peak at 1641 cm^−1^ is the bending vibration of –OH, and 1300–1000 cm^−1^ corresponds to the stretching vibration of C–O–C [30], indicating that adding nano-SiO_2_ did not disrupt the molecular structure of the cellulose. The FTIR spectrum of the nano-SiO_2_ in Figure 3a indicates characteristic peaks at 1068 and 802 cm^−1^, attributable to Si–O–Si antisymmetric stretching vibrations and Si–O symmetric stretching, respectively [24]. Compared with the RC0 film, the infrared spectrum of the RC6 film indicates new absorption peaks at 849 and 1233 cm^−1^, corresponding to the bending vibration of Si–O and C–Si, respectively, indicating the mixing and strong interactions between the nano-SiO_2_ and cellulose chains [31]. In addition, the OTS’s distinctive absorption peak was recorded at 2850 cm^−1^, which confirms the modification of the RC films with the OTS.

Figure 3b shows the XRD diffraction patterns of the wood pulp cellulose, RC0 film, RC6 film, and HRC2 film. The wood pulp cellulose exhibited a typical cellulose I crystalline form, with peaks at 16.0°, 22.6°, and 34.6°. These peaks corresponded to the (110), (110), and (040) planes, respectively. A total of three typical crystal peaks of the RC0, RC6, and HRC2 films at 2θ = 12.0°, 20.0°, and 21.0° were attributed to the crystal planes (110), (110), and (200), respectively, of cellulose II [32]. Most of the other cellulose solvent solutions followed a similar trend [33,34,35,36]. Furthermore, the minor diffraction peak at roughly 35° corresponded to a plane with a Miller index of (004). The degree of crystallinity of the wood pulp cellulose was calculated from the diffractograms to be 50.2%, and that of the RC0, RC6, and HRC2 films was 23.3%, 21.14%, and 19.3%, respectively. These results indicate that strong hydrogen bonding interacting nano-SiO_2_ and cellulose chains break the ordered arrangement of cellulose molecular chains, leading to the crystalline region of the cellulose to shrink while the amorphous region expands, making these RC films less crystalline than the RC0 film [37]. In comparing the diffraction curves of the RC0, RC6, and HRC2, all of the peaks in the RC0 were in the spectra of the RC6 and HRC2. The addition of the SiO_2_ and surface silanization had little effect on the RC film’s crystal structure.

Figure 3c presents the solid ^13^C-NMR spectra of the cellulose, RC0 film, RC6 film, and HRC2 film that were measured in this system. The chemical shifts of the cellulose were 105.1 ppm for C1, 88.6 ppm for C_4_, 74.6 ppm and 72.4 ppm for C_2,3,5_, and 65.1 ppm for C_6_. C_4_ and C_6_ had strong and sharp signal peaks at 88.6 ppm and 64.9 ppm, broadly and relatively. The signal peaks at 83.0 ppm and 61.9 ppm were broadly and relatively weak, showing the crystalline and amorphous regions of the cellulose, respectively [38]. The chemical shifts of the RC film were 104.7 ppm, 83.1 ppm, 74.8 ppm, and 62.7 ppm for C_1_, C_4_, C_2, 3,5,_ and C_6_, respectively. Compared with the cellulose, the C_4_ of the RC film showed almost no signal at 88 ppm and an enhanced signal peak at 83 ppm, indicating that the hydrogen bonding network was broken during the dissolution process, that the cellulose crystalline region was reduced, and that the amorphous region increased; the C_6_ that shifted from the cellulose was 64.9 ppm to 62.7 ppm, and changed from a duplex peak to a single peak, indicating that the hydrogen bonding network of the sample was broken and that the hydroxyl group conformation at the C_6_ position changed from cellulose I to regenerated cellulose II [39]. The NMR signal peak near the chemical shift value of 33.2 ppm in the HRC film carbon nucleogram was C–Cl, which indicated the successful grafting of the OTS with the cellulose.

**Figure 4:**
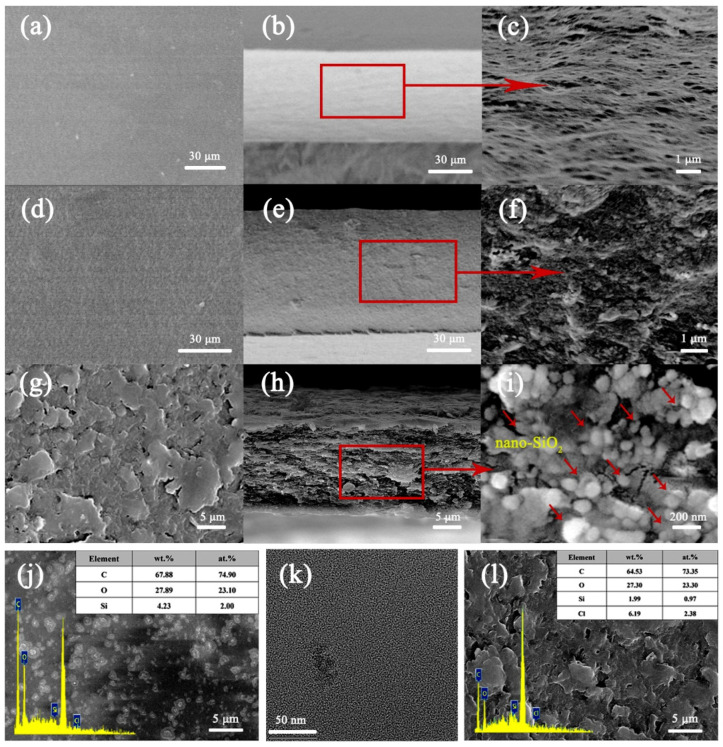
SEM images of the top surface and cross-sectional surface of the (**a**–**c**) RC0, (**d**–**f**) RC6, and (**g**–**i**) HRC2 films at various magnifications. EDS elemental analyses of the (**j**) RC6 and (**k**) HRC2 films. TEM image of (**l**) the RC6 film.

According to the SEM image of the RC0, the surface of the RC0 film was smooth and flat, and the cross-sectional structure was uniform and dense (Figure 4a–c). Compared with the RC0 film, Figure 4e depicts the SEM pictures of the RC6 film’s fractured surface. The surface of the RC6 film became rough and white particles were dispersed, which differs from the fractured surface of the original film. The surface roughness of the film is related to the increased nano-SiO_2_, and the micro–nano scale rough surface structure imparts a higher WCA to the surface of the RC film. In Figure 4f, at a higher magnification, the nanoparticles filled the pores, indicating that there were good interfacial interactions between the nano-SiO_2_ and the cellulose; the nano-SiO_2_ enhanced the tensile strength of the RC film [40]. The dense structure of the composite film increased its mechanical qualities. In the enlarged cross-sectional view, dispersed nano-SiO_2_ particles were evident without an obvious agglomeration. The TEM observations indicated that the nano-SiO_2_ (white crystals) was uniformly distributed in the cross-section of the film (Figure 4k). Through the SEM imaging of the nano-SiO_2_/cellulose composite film after incorporating the OTS (HRC), it was evident that a rough layered structure formed on the surface, rougher than that of the RC film, indicating that the incorporation of the OTS could increase the film’s performance. The hydrophobic properties of the HRC films suggest that the hydrophobic alkyl, silicon, and chlorine elements that were introduced by the hydrophobic modification underwent graft copolymerization onto the surface of the composite film. The pores became smaller. With the increasing quantity of the OTS, more hydrophobic alkyl groups were introduced and more positions were occupied, and the pores gradually became smaller. The pores were evident in the cross-sectional structure, were insufficiently dense, and affected the mechanical properties of the HRC films. According to the EDS images, the RC6 film contained C, O, and Si (Figure 4j), and the HRC2 film contained C, O, Si, and Cl (Figure 4l). The percentage of each element in Figure 4 represents the elemental composition of the test part.

### 3.2. Mechanical Properties and Thermal Stability

**Figure 5:**
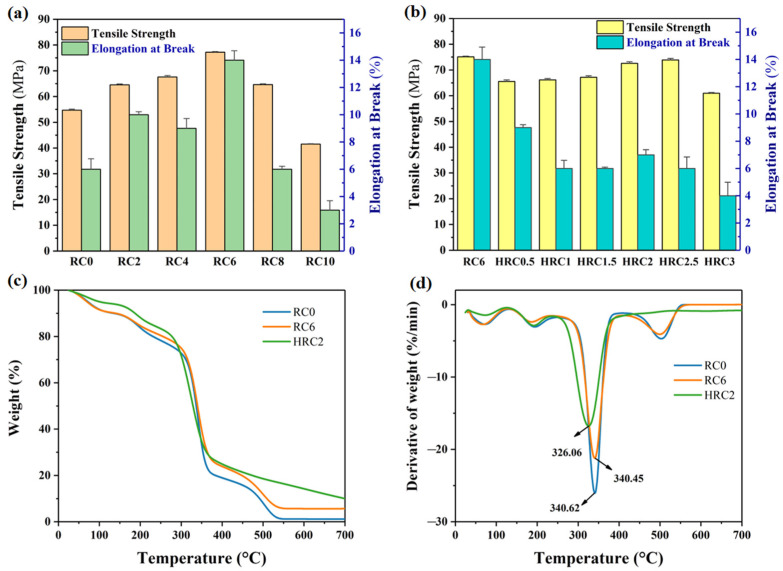
Tensile properties of the (**a**) RC, and (**b**) HRC films. (**c**) TGA thermograms, and (**d**) differential thermograms of the RC0, RC6, and HRC2 samples.

The mechanical characteristics of the nano-SiO_2_/regenerated cellulose composite films were compared to those of the RC0 film to further reinforce the conclusion that the nano-SiO_2_ particles exhibited significant interfacial contact with the cellulose chains. Figure 5c indicates that by increasing the nano-SiO_2_ from 0 to 6 wt%, the tensile strength of the cellulose films increased from a value of 54.69 MPa to a value of 77.22 MPa (41.2%), and the elongation at break increased from a value of 5.9% to a value of 14% (57.86%); the tensile strength and elongation at break of the RC6 were the highest. However, when the nano-SiO_2_ exceeded 6 wt% (Figure 5a), the composite films’ tensile strength and elongation at break decreased as the nano-SiO_2_ concentration increased. A similar phenomenon has been seen in cellulose/carbon nanotube composite fibers [41], cellulose/graphene oxide composite films [42], and other polymers/SiO_2_ composites (including polypropylene/SiO_2_, polyamide6/SiO_2_, and polyimide/SiO_2_), attributable to the nano-SiO_2_ particles’ greater propensity to form agglomerates and impair material homogeneity at high concentrations [43,44,45,46], which decreases the performance and eventually fails to retain the characteristics of the nanoparticles. In addition, because of the differences in the structure of the inorganic particles and organic phases, the nano-SiO_2_ particles did not uniformly disperse in the matrix, which deteriorated the performance of the RC films. All of the RC films had a better tensile strength than the RC0 film (due to the strong hydrogen bonding interactions between the nano-SiO_2_ and cellulose chains), and did not exhibit a strong agglomeration of the nano-SiO_2._

The hydrophobically modified HRC films with the OTS/hexane still have an excellent tensile strength and elongation at break, which can meet the technical requirements of the national standard GBT22781-2008 for moisture-proof cellophane (Figure 5b). Therefore, the preparation of the hydrophobic HRC films—with improved mechanical properties—will facilitate the production of cellulose-based, high-performance materials.

Figure 5a,b compares the thermal stability of the RC0, RC6, and HRC2; the heating temperatures of the RC0, RC6, and HRC2 at a 20% weight loss were 230.89 °C, 255.2 °C, and 277.23 °C, respectively. When the weight loss was 80%, the heating temperatures were 383.64 °C, 447.95 °C, and 473.57 °C. Compared with the RC0 film, the RC6 had a higher stability than the RC0 film at temperatures less than ca. 330 °C (Figure 5c). Further comparison of the DTG curves shows that, with the addition of SiO_2_, both the initial thermal degradation temperature and the thermal degradation peak temperature of the RC6 film almost coincided (Figure 5d). The TGA curve indicates that, at 700 °C, the residual mass of the RC6 film was 0.474 g; the increase of the residual was nearly equal to the quantity of the nano-SiO_2_ integrated into the cellulose, indicating that the nano-SiO_2_ had a high thermal stability due to the regular tetrahedral mesh structure imparting a good heat resistance to the silica.

### 3.3. Optical and UV-Blocking Properties of the RC Films and HRC Films

**Figure 6:**
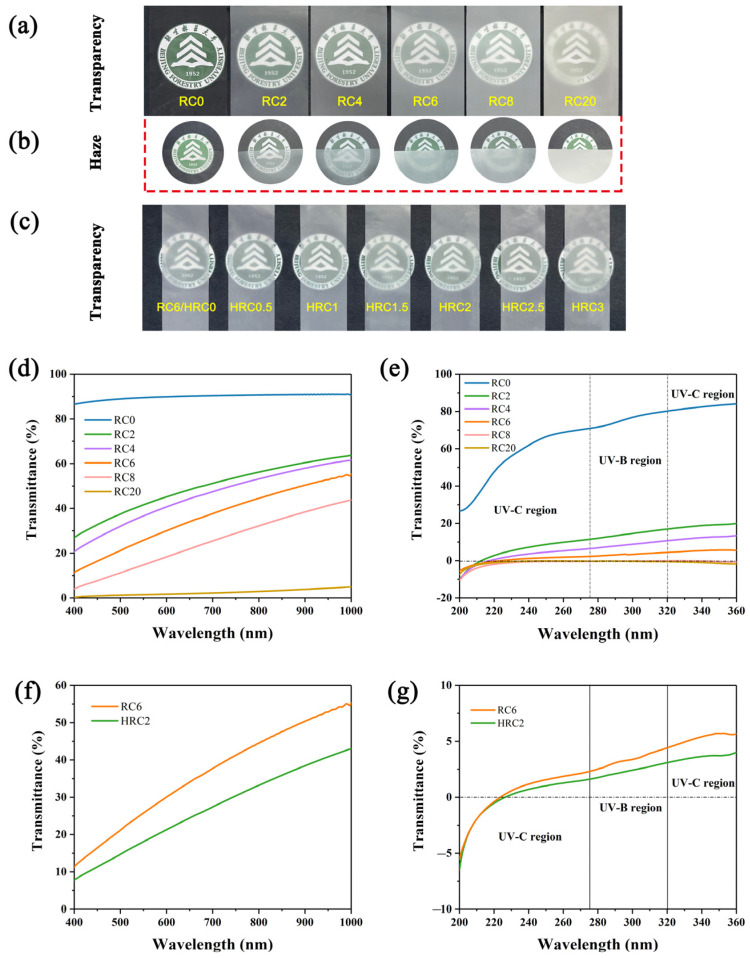
(**a**) Photos of the patterns recorded through closed, covered RC films, (**b**) the patterns taken with the RC films with 1 cm higher, and (**c**) photos of closed, covered HRC films. UV–vis spectra of the (**d**,**e**) RC, and (**f**,**g**) HRC2 films.

Figure 6 shows the optical performance of the RC and HRC films. The transparency and haze were first evaluated via digital photos. Digital images were used to assess the transparency and haze. The transparency of the films was decreased and the haze was enhanced with an increasing dosage of SiO_2_. The pattern appeared indistinct when the RC20 film was held 1 cm above the backdrop, due to significant light scattering, which showed considerable optical haze. The background pattern 1 cm distant from the RC0 film, on the other hand, remained distinct, demonstrating its ultralow haze and exceptional transparency (Figure 6a,b). The morphology and microstructure of the films, in general, are significant elements that influence their optical properties. Moreover, the transparency and haze of the HRC films exhibited no substantial changes, suggesting that the surface modification had only a minor effect on the optical characteristics of the film. (Figure 6c).

The optical properties of the RC films were further evaluated by UV−vis spectroscopy (Figure 6d,e). The transparency of the five composite films grew steadily from UV to visible light. The transmittance of the RC2 was up to 41.6% over this wavelength range, indicating that the nanocomposite films that were developed in this work have prospective uses in packaging and covering films [47,48,49,50]. At a 550 nm wavelength, the transmittance of the RC films with 0, 2, 4, 6, and 8 wt% nano-SiO_2_ was 89.5%, 41.6%, 36.6%, 25.8%, and 14.8%, respectively. The transmittance of the RC film decreased substantially when the nano-SiO_2_ content exceeded 8%. SiO_2_ might facilitate the creation of hydrogen bonds between the cellulose molecular chains. The hydrogen bonding affected the visible light transmittance of the composite films.

Most of the UV radiation between 200 to 400 nm could be virtually fully absorbed by the transmittances of the RC films with various contents of SiO_2_. Figure 6f,g shows the UV-blocking rate for the HRC2 film. The HRC2 film blocked approximately 100% of the UVB and over 95% of the UVA, which was much greater than the RC film’s blocking. The superior UV blocking performance of the HRC2 film over the RC6 film suggests that the OTS/*n*-hexane modified surface had a beneficial influence on the film’s UV blocking performance. According to the aforesaid findings, the composite films show promising possibilities for UV-blocking applications in packaging and coating.

**Figure 7:**
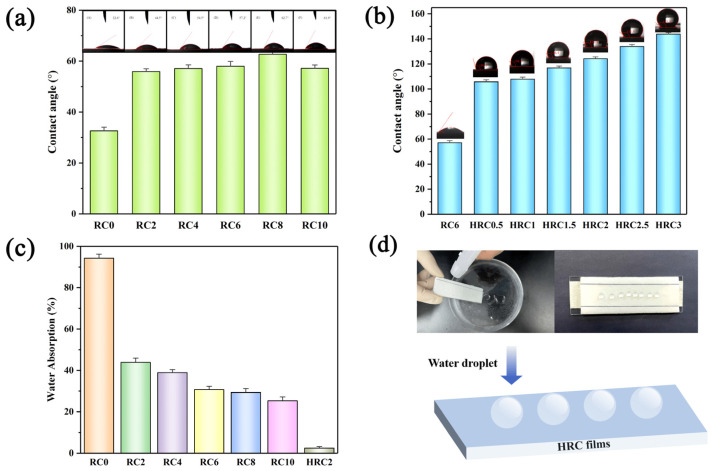
Water contact angles of the (**a**) RC, and (**b**) HRC films. Water adsorption of the (**c**) RC and HRC2 films. (**d**) Schematic of the hydrophobic surface of the HRC films, and an image of water droplets’ self-cleaning dust on the HRC films.

The WCA indicates a material’s hydrophilic/hydrophobic characteristics [47,48]. Figure 7a,b shows the WCA of the films. Commonly, an increasing hydrophobicity of a material corresponds to a lower contact angle. The WCA of the RC0 film was 36.2° ± 0.3° (Figure 7a), indicating a hydrophilic film, which limits its applications in humid conditions. However, the contact angle of the RC films increased gradually with an increasing nano-SiO_2_ content; the contact angle of the RC8 film was 62.7° ± 0.5° (Figure 7a). This result indicates that incorporating nano-SiO_2_ into the RC films substantially improved the films’ hydrophobicity. The reason for this increased hydrophobicity might be two-fold: first, the RC films’ higher surface roughness in comparison to the RC0 films (Figure 7a) [50,51,52], and second, a water-impermeable barrier being formed by the strong hydrogen bonding between the nano-SiO_2_ and the cellulose [53]. When the nano-SiO_2_ content exceeded 8%, the WCA of the RC10 decreased. This result is because of the agglomeration of the nano-SiO_2_ on the surface of the composite film, which enhanced the surface hydrophilicity.

The hygroscopicity test was used to indicate the water absorption, in order to further confirm the water stability of the composite films. After immersing the samples in water for over 24 h, the water absorption of the RC0 was ca. 104.3% upon reaching equilibrium. After blending, the water absorption of the films decreased from a value of 43.9% (RC2) to a value of 25.3% (RC8) with an increasing nano-SiO_2_ content (Figure 7c). The water sorption in cellulose is primarily mediated by free hydroxyl groups in amorphous areas [30,49]. Furthermore, the nano-SiO_2_ particles have silanol hydroxyl groups of Si-OH on their surfaces; these functional groups have strong hydrogen bonding interactions with the free hydroxyl groups (–OH) in cellulose chains. and Si–CH_3_ reduces the hydrophobicity of the composite films [54,55]. Therefore, adding nano-SiO_2_ to cellulose films can minimize their water absorption. In other words, the nano-SiO_2_/regenerated cellulose composite films outperformed the RC0 film with regard to water resistance.

After the surface hydrophobic modification, the water absorption of the HRC2 film was reduced to 2.4% (Figure 7c), which is because low-surface-energy substances act as a water-resistant buffer on surfaces, resulting in a substantially improved water resistance [56]. Figure 7b indicates that the hydrophobicity of the RC films depended on the dosage of the OTS/*n*-hexane hydrophobic agent. After the surface modification, the HRC films exhibited a sharp increase in the WCA compared with that of the unmodified RC6. The average WCA of the obtained HRC films increased from a value of 105.75° ± 0.6° for the HRC0.5 film to 143.8° ± 1.0° for the HRC3 film (Figure 7b). Figure 7d shows a photo that enables a comparison of the RC6 before and after the surface modification with the OTS/*n*–hexane hydrophobic agent. The hydrophobic film indicates that the water droplets slid off the surface of the HRC3 rapidly (Video S1), demonstrating its outstanding hydrophobicity. In principle, these phenomena are attributable to the fact that both the regenerated cellulose matrix and the nano-SiO_2_ surface were silanized during the graft modification of the composite film surface by the OTS, which reduced the surface energy and increased the hydrophobicity of the composite film [57].

### 3.4. Oxygen Barrier Properties of the RC and HRC Films

**Figure 8:**
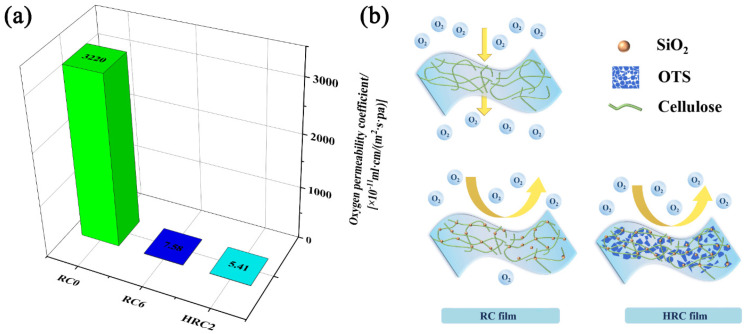
(**a**) Oxygen permeability of the RC0, RC6, and HRC2 films. (**b**) Schematic representation of SiO_2_ and OTS morphologies (and gas permeating path) in the RC0, RC6, and HRC2 films.

Inorganic nanoparticles are often used as oxygen scavengers to reduce the oxygen transmission rate of packaging materials [42,57]. Figure 8a indicates that, under a 50% relative humidity, the oxygen permeation rate of the RC0 film was 1.11 × 10^4^ mL/(m^2^·s·Pa), and the oxygen permeability coefficient was 3.22 × 10^−8^ ml·cm/(m^2^·s·Pa). The oxygen permeability coefficient indicates the properties of the polymer material and does not include the effect of the film thickness on the oxygen barrier properties. The RC0 film had a high oxygen permeability; the oxygen molecules gradually passed through the nanopores in the RC film, and thus the oxygenation performance of the film required further improvement. The addition of the nano-SiO_2_ substantially improved the RC films’ oxygen barrier properties. The oxygen permeability coefficient of the RC6 film was 7.58 × 10^−11^ mL·cm/(m^2^·s·Pa). The inorganic nanoparticles filled the matrix’s holes and reduced the porosity of the polymer, yet hindered the vertical penetration of the oxygen. Thus, the penetration path was convoluted in a manner that the oxygen migration rate was lowered; hence, this increased the polymer’s barrier properties. The oxygen permeability coefficient of the HRC2 film was 5.41 × 10^−11^ mL·cm/(m^2^·s·Pa). The OTS was introduced into the regenerated cellulose matrix for the surface silanization graft modification, in order to improve the oxygen barrier performance, for two main reasons. First, the surface layered structure can block the diffusion of oxygen from all directions and increase the diffusion path of the oxygen, because oxygen molecules cannot directly pass through the surface of the composite film. Second, the OTS has a large specific surface area. When molecular oxygen diffuses, it is adsorbed onto the surface of the OTS sheet. At this time, the oxygen molecule fills the space between the OTS and the regenerated cellulose gaps, resulting in the HRC film’s low oxygen permeability (Figure 8b).

The oxygen permeability coefficient of the HRC film was 0.01% lower than that of the RC0 film, all of which reached the oxygen barrier requirements of GB-T30768-2014 food packaging paper and plastic composite films and bags. Therefore, the manufactured RC films can be employed as a packaging material to preserve perishable commodities from oxygen damage, which can mitigate the oxidation of food and facilitate prospects of food preservation.

## 4. Conclusions

In this study, an environmentally friendly solvent system was used to quickly dissolve cellulose at room temperature. The incorporation of nano-SiO_2_ as a nanofiller in a pure cellulosic material significantly enhanced the mechanical properties of the RC films, and resulted in an excellent UV-shielding ability and oxygen resistance. After the surface silanization modification, the obtained HRC films exhibited a hydrophobic surface (HRC3 WCA = 143.8°) with a self-cleaning function that mitigated dust accumulation. The UV resistance and oxygen barrier properties of the HRC films were also improved. The HRC2 film especially indicated the potential to block almost all UV rays over the entire UV region (200–400 nm). An environmentally friendly preparation technology of fully biodegradable materials was developed to manufacture high-strength, UV-resistant, gas-blocking, biodegradable RC films, which have potential applications in packaging and functional materials.

## Data Availability

Data is contained within the article or Supplementary Material. The data presented in this study are available in insert article or Supplementary Material here.

## Supplementary Materials

The following supporting information can be downloaded at: https://www.mdpi.com/article/10.3390/polym15061427/s1, Table S1: The results of tensile tests of RC films and HRC films.; Table S2: The zero-shear rate viscosity of composite film liquid with different addition of nano-SiO_2_ at 25 °C; Table S3: Weight loss rate of the RC0 in soil; Figure S1: Changes of the RC0 during soil degradation; Video S1: Water droplets quickly roll away on the surface of the HRC2 [58].
