# Supplementary material for: Surface Engineering of Regenerated Cellulose Nanocomposite Films with High Strength, Ultraviolet Resistance, and a Hydrophobic Surface
Source: Polymers (Basel). 2023 Mar 14;15(6):1427. doi: 10.3390/polym15061427 (PMC10053694; doi:10.3390/polym15061427)
Supplement: Supplementary file 1 [file polymers-15-01427-s001.zip › Supplementary Material.pdf]

Supplementary Material

Surface Engineering of Regenerated Cellulose Nanocomposite Films with High Strength, Ultraviolet Resistance, and a Hydrophobic Surface

Ying Zhu, Tianhao Wang, Yanan Dai, Ye Wang, Yukun Ding, Liping Zhang*

Department of Chemistry and Chemical Engineering, MOE Engineering Research Center of Forestry Biomass Materials and Bioenergy, Beijing Forestry University, Beijing 100083, China.

***Corresponding Authors**

Liping Zhang - Department of Chemistry and Chemical Engineering, Beijing Forestry University, Beijing, 100083, China; Email: zhanglp418@163.com; Tel/Fax: 010-62338152

Mechanical Properties of RC films and HRC films

Table S1. The results of tensile tests of RC films and HRC films.

Sample	Thickness (μm)	Tensile Stress (N)	Tensile Strength (MPa)	Tensile Strain at Fracture (%)	Gram Weight (g/m^2)
RC0	32±0.82	26.25±0.41	54.69±0.41	6±0.77	40.69
RC2	29±0.82	28.08±0.36	64.55±0.36	10±0.22	38.26
RC4	29±0.5	29.40±0.58	67.59±0.58	9±0.72	37.97
RC6	30±0.5	33.81±0.27	77.22±0.27	14±0.9	39.45
RC8	29±0.47	28.12±0.28	64.64±0.28	6±0.22	38.10
RC10	30±0.83	18.690±0.14	41.53±0.14	3±0.69	40.34
HRC0.5	30.25±0.88	29.74±0.48	65.54±0.61	9±0.21	40.87
HRC1	31.5±0.71	31.26±0.77	66.16±0.53	6±0.59	42.28
HRC1.5	30.25±0.71	30.48±0.5	67.17±0.56	6±0.09	40.29
HRC2	30±0.35	32.66±0.77	72.58±0.63	7±0.37	39.93
HRC2.5	30.75±0.11	34.09±0.19	73.91±0.58	6±0.84	41.125
HRC3	30.5±0.35	27.9±0.24	60.98±0.26	4±0.99	41.29

Rheological properties of composite film liquid

Table S2. The zero-shear rate viscosity of composite film liquid with different addition of nano-SiO_2 at 25 °C.

Sample	η_0 (Pa·s)
RC0	252.8
RC2	283
RC4	324.2
RC6	339.9
RC8	258.2

Biodegradable behaviors of the RC0

Table S3. Weight loss rate of the RC0 in soil.

Time (d)	0	3	6	9	12	15
Weight loss rate (%)	0	10.95	18.57	23.10	45.71	100

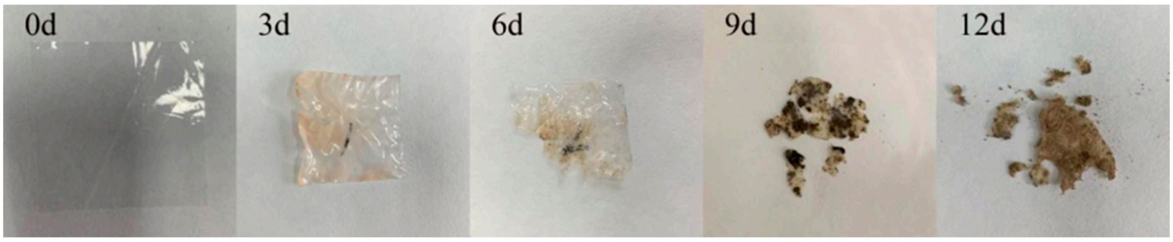

Figure S3. Changes of the RC0 during soil degradation.

From the perspective of environmental protection, biodegradability is the basic requirement of packaging materials.[1] As shown in Figure S3, RC0 became some cracks after burying in soil for 3 days. The fragmentation process of RC0 during its biodegradation in soil could be found in Figure S3. This phenomenon can be explained by the fact that the film was attacked and digested by the microorganisms in the soil. Furthermore, some fungal mycelia began to appear on the surface after 3 days of degradation, and a large number of fungal mycelia appeared on the film after 6 days. The microorganisms and broken fragments of RC0 were observed after 9 days. Table S2 shows the course of weight loss against degradation time for the RC0 film buried in the soil. From the extrapolation of the plots, RC0 could be biodegraded completely by the microorganisms in the soil at 32 °C after 15 days. Compared to the commercial plastics, these regenerated cellulose films were safe and biodegradable after being used.

References:

[1] K. Zhu, H. Tu, P. Yang, C. Qiu, D. Zhang, A. Lu, L. Luo, F. Chen, X. Liu, L. Chen, Mechanically Strong Chitin Fibers with Nanofibril Structure, Biocompatibility and Biodegradability, CHEM MATER (2019).
